# Same-Sex Relationships and Criminal Behavior: A Total Population Study in The Netherlands

**DOI:** 10.1007/s10508-024-02902-9

**Published:** 2024-06-21

**Authors:** Steve G. A. van de Weijer, Sjoukje van Deuren, Brian B. Boutwell

**Affiliations:** 1 Netherlands Institute for the Study of Crime and Law Enforcement, PO Box 71304, 1008 BH Amsterdam, The Netherlands; 2 Department of Criminology, VU University Amsterdam, Amsterdam, The Netherlands; 3 School of Applied Sciences, The University of Mississippi, Mississippi, MS USA; 4 John D. Bower School of Population Health, University of Mississippi Medical Center, Mississippi, MS USA

**Keywords:** Sexual orientation, Crime, Same-sex union, Discordant siblings

## Abstract

Sexual minority groups experience elevated risk across a range of adverse outcomes. Previous studies from the USA showed that these risks include contact with the criminal justice system for sexual minority females but not for males. This study examined whether this relationship between sexual minority status and criminal behavior was also found in a more secular country like the Netherlands with more progressive attitudes toward sexual minorities. Furthermore, the study aimed to examine whether this relationship applied to various types of crime and could be explained by unmeasured familial factors. Longitudinal data from the Dutch national population, including 75,362 individuals in a same-sex relationship and 3,464,906 individuals in opposite-sex relationships, were used to compare the risk of crime among males and females in same-sex and opposite-sex unions. Discordant sibling models were included to increase control over possible sources of confounding from shared familial factors. Analyses were repeated for six types of crime, including property offenses, violence, vandalism, and public order offenses, traffic offenses, drugs offenses, and other offenses. The results showed that the direction of the associations between same-sex relationships and offending differed for men and women. In general, men in same-sex relationships were less likely to be a suspect of crime compared to those in opposite-sex relationships [odds ratio (OR) = 0.685; *p* < .001]. Women in same-sex relationships exhibited higher risk than those in opposite-sex unions (OR = 1.560; *p* < .001). Similar patterns emerged for most crime types and the discordant sibling models yielded conclusions that were substantively similar to those among the total population.

## Introduction

A considerable amount of evidence suggests that non-heterosexual individuals are disproportionately exposed to various types of adversity (Kiekens et al., 2021). Systematic reviews and meta-analyses have consistently shown that non-heterosexual individuals have an elevated risk of abuse in childhood (Friedman et al., 2011), other forms of criminal victimization with age (Katz-Wise & Hyde, 2012; Myers et al., 2020; Toomey & Russel, 2016), vulnerability to substance use (Goldbach et al., 2014; King et al., 2008; Marshal et al., 2008), elevated risk of psychiatric problems (King et al., 2008; Semlyen et al., 2016), as well as an increased vulnerability to suicidal behaviors (King et al., 2008; Miranda-Mendizábal et al., 2017).

Antisocial and criminal behaviors constitute another source of risk for social adversity for non-heterosexual individuals, and there is relatively robust evidence that it covaries with each of the risk factors mentioned above (Buttar et al., 2013). This raises the possibility that sexual orientation itself may covary with antisocial behaviors. Some nascent evidence suggests that there is a correlation, but efforts to replicate this initial work have remained limited. Decades ago, some indications emerged suggesting that lesbians were, for example, more likely to be involved in physical aggression and crime compared to heterosexual females (Ellis et al., 1990; Pinhey & Brown, 2005). At earlier life-course stage, evidence emerged that women identifying as a sexual minority group member, such as lesbians or bisexual women, were disproportionally involved in the juvenile justice system in six out of seven studies (Belknap et al., 2014; Buttar et al., 2013; Himmelstein & Brückner, 2011; Hirschritt et al., 2018; Irvine, 2010; Irvine & Canfield, 2016; Wilson et al., 2017) included in the systematic review of Jonnson et al. (2019). The pooled prevalence of sexual minority girls in custody in these studies was about twice as high as community estimates. A different pattern of findings emerged for the relationship between sexual minority status and crime among males. Gay males appear to display lower levels of physical aggression and criminal behavior as compared to heterosexual males (Ellis et al., 1990; Pinhey & Brown, 2005; Sergeant et al., 2006). Furthermore, sexual minority boys, in contrast to sexual minority girls, did not appear to be overrepresented in the juvenile justice system (Jonnson et al., 2019). Though limited, at least some early evidence suggested that bisexual males tended to report more involvement in violence and crime than heterosexual males (Ellis et al., 1990).

Similar associations between sexual orientation and delinquency were found in a large national sample of American respondents (Beaver et al., 2016). Bisexual respondents reported the highest levels of involvement in delinquent behavior, followed next by heterosexual males, lesbians, gay males, and heterosexual females. Arguably the most noteworthy finding was that lesbian respondents seemed to be more involved in crime than gay males (Beaver et al., 2016). The novelty here stems from the fact that one of the most replicated findings in behavioral science is sex-differentiated criminal involvement across virtually all crime types (Ellis et al., 2009; Moffitt et al., 2001).

Beyond the epidemiological evidence pointing to a relationship between sexual orientation and antisocial or criminal behavior, certain theoretical perspectives related to aspects of sexual orientation have also offered reason to suspect that such a relationship could exist. To date, however, no clear frontrunner seems to have emerged. First, biologically oriented theories, the prenatal androgen theory in particular (Ellis & Ames, 1987), offer explanations for links between sexual minority status and crime (Lippa, 2020). The prenatal androgen theory suggests that gay males and lesbians are exposed to atypical levels of prenatal testosterone compared to heterosexual individuals (Ellis & Ames, 1987). On average, gay males are exposed to lower prenatal testosterone levels than heterosexual males, while lesbians are exposed to higher prenatal testosterone levels than heterosexual females. What the theory essentially describes is a type of “*cross-gender shift,*” such that gay males display more traditionally “feminine” traits, and lesbians appear more “masculine” relative to their heterosexual counterparts.

Central to the emergence of this shift is the role of testosterone, thought to be impactful in both the formation of sexual identity early in development and the regulation of behavior later in the life course (Book et al., 2001; Ellis, 2005). Drawing on this perspective, one may expect that rates of crime should be lower for gay males and higher for lesbians, a prediction with some support (Beaver et al., 2016; Ellis et al., 1990; Johnson et al., 2019; Pinhey & Brown, 2005). That aside, the broader success of the theory hinges on predictions about testosterone specifically that, so far, appear far less compellingly supported by evidence. The general effects of testosterone on antisocial and aggressive outcomes typically reach statistical significance, but clinically speaking, the effect sizes are weak (Book et al., 2001). Evidence pertaining to links between prenatal testosterone levels and either sexual orientation or antisocial behavior exists in a largely similar state (Breedlove, 2017). Meta-analyses have revealed some support for the effects on antisocial behaviors, but they too were decidedly weak in magnitude, challenging the core tenets of the theory (Pratt et al., 2016; Turanovic et al., 2017).

A second explanation offered for the relationship between sexual orientation and crime is derived from the minority stress model. This model proposes that prejudicial and discriminatory cultures create hostile and stressful social environments for sexual minority group members, thereby increasing their risk for various deleterious outcomes (Lick et al., 2013; Meyer, 2003). This includes an increased risk for antisocial and criminal behavior since victimization (Jennings et al., 2012) and psychological problems in general (Hodgins et al., 1996; Joyal et al., 2007) are robust correlates of such behaviors. Victimization and perpetration in particular are so tightly linked that criminologists rarely dispute the connection and refer to it as “victim-offender overlap” (Berg & Schreck, 2022; Jennings et al., 2012). It remains less clear, however, whether sexual minority group members who have an elevated risk of victimization also evince elevated risks of perpetration. Something to keep in mind, too, is that based on the theory, we would expect uniform increases in offending across groups. So far, this prediction has not fared well, but it has also received relatively little empirical attention. Recall that prior work has reported increased levels of antisocial behavior among bisexuals and lesbians but not among gay males (Beaver et al., 2016), a result that is more in line with biologically oriented theories that predict a cross-gender shift than with the minority stress model.

The central motivation of this study was to further explore the relationship between sexual minority status and criminal behavior by examining longitudinal population wide register data from the Netherlands. Several qualities make these data highly appealing, and we discuss those below. But there is an important caveat that needs to be mentioned first concerning a key limitation when using the register to test this particular issue. The nature of the data do *not* permit us to directly assess sexual identities and orientations as reported by individuals in the sample. We cannot, for example, distinguish individuals identifying as gay, from those identifying as bisexual, and so on.

What we rely on instead is a proxy measure consisting of official registrations (i.e., marriage or registered partnership) of same-sex and opposite-sex relationships (see also Xu et al., (2023) for a similar approach). This approach therefore captures the types of formal actions (e.g., marriage) taken by individuals when choosing partners based on sexual identity and romantic preferences. In this study, men and women who are or ever have been in a same-sex relationship are considered as a sexual minority group member, either gay or lesbian. Individuals who have only ever had opposite-sex relationships are considered to identify as heterosexual. That said, in order to keep this important point about our data present in the mind of readers, when discussing our results, we refer to either men or women in either “same-sex” or “opposite-sex” unions, and not to them as belonging to a particular sexual minority group or orientation.

This study is specifically intended to fill three areas with limited clarity. First, existing research predominantly measured criminal involvement in general (Jonnson et al., 2019) or only distinguished violent from non-violent antisocial behaviors (Beaver et al., 2016). It remains largely unknown whether men and women in same-sex relationships are over- or underrepresented for all or only certain types of crime. We distinguish between six types of crime (i.e., property offenses, violence, vandalism and public order offenses, traffic offenses, drugs offenses, and other offenses) to better explore this issue.

Second, because of certain methodological limitations, there are lingering concerns about omitted variables biasing the calculation of effect sizes in prior work. Like many topics in behavioral science, studies conducted on aspects of human sexuality and mating choice are rarely able to capitalize on experimentation (see Barnes et al., 2014; Willoughby et al., 2023). However, certain designs can help facilitate more rigorous corrections for spurious influences in observational data analysis (e.g., Barnes et al., 2014; D’Onofrio et al., 2013; Gonggrijp et al., 2023; Willoughby et al., 2023). By utilizing a discordant sibling model, individuals in a same-sex relationship are compared to their sibling in an opposite-sex relationships. By comparing within sibling pairs rather than between unrelated individuals, this model accounts for environmental influences shared between siblings, while partially correcting for genetic influences that might confound associations (D’Onofrio et al., 2013; Willoughby et al., 2023). This is particularly important because indicators of human sexuality (Bailey & Pillard, 1995; Cook, 2021; Kendler et al., 2000; Kirk et al., 2000; Zietsch et al., 2021) and antisocial behaviors (Ferguson, 2010; Mason & Frick, 1994; Rhee & Waldman, 2002) have heritability estimates above zero. The practical implication of this is that genetic factors join environmental factors as possible confounders if left uncorrected.^1^ The variety of sibling-based modeling used here can offer at least partial corrections for genetic influence, whereas standard analyses using unrelated individuals cannot (Barnes et al., 2014; Willoughby et al., 2023).

Third, to the our knowledge, only one study so far has examined sexual minority involvement in the justice system outside of the USA (i.e., Canada; Smith et al., 2013). There is a need to explore the possibility that the results of prior work emerged as a product of cultural and societal forces idiosyncratic to the United States. It is possible that social forces that are either less prevalent or absent entirely in other cultures could impact efforts to replicate previous findings. The Netherlands has a largely secular population (Statistics Netherlands, 2022) and was the first country to legalize same-sex marriages, in 2001. This matters, because as the results of Van den Akker et al. (2013) suggested, the degree of religiosity and laws pertaining to sexuality in a country are relevant factors for understanding the presence of widespread disapproval of alternative sexual identities. It is less surprising, then, that the Netherlands is among countries with the most positive and progressive attitudes toward sexual minorities, but it is unclear how this might impact overt behaviors of individuals differing in their sexual interests (Smith et al., 2014; Van den Akker et al., 2013).

Cultural attitudes and acceptance matter from a theoretical standpoint, in addition to its possible methodological implications. Individuals in same-sex relationships in the Netherlands may experience less stigma, prejudice, and discrimination than those in countries with less progressive attitudes toward sexual minorities. For biologically informed theories, this should present with only minor consequences for behavioral outcomes, as the crucial variable is considered to be hormone exposure in utero. From the vantage point of the minority stress model, we might expect that associations between same-sex relationship status and antisocial behaviors would become attenuated as societies grow more progressive and inclusive. The reason why, of course, is that cultural forces, such as prejudice and bias, are considered to represent the key upstream source of psychological distress that might ultimately manifest as antisocial behavior.

### The Current Study

By addressing the gaps of knowledge discussed above, the current study will answer three research questions. First, is an association between having a same-sex relationship and criminal behavior also found in a more secular country like the Netherlands with more progressive attitudes toward sexual minorities than the USA? Second, is this association the same for various types of crime? And third, does this association remain significant after accounting for unmeasured familial confounders?

Based on the minority stress model, it can be hypothesized that both men and women in same-sex relationships are more likely to offend than those in opposite-sex relationships. On the other hand, based on biologically oriented theories, such as the prenatal androgen theory, it can be hypothesized that women in same-sex relationships are more likely to offend than women in opposite-sex relationships, while men in same-sex relationships are less likely to offend than men in opposite-sex relationships.

## Method

### Sample

Register data from Statistics Netherlands, a Dutch governmental institution that gathers statistical information about the Netherlands, were utilized in the current study.^2^ These data contain various types of official information on all Dutch inhabitants which can be linked through an anonymized identification number and is updated annually. Most data used in the current study were obtained from the Personal Records Database (in Dutch: BasisRegistratie Personen; BRP), which has complete coverage of all Dutch inhabitants from 1994 onwards. Same-sex and opposite-sex couples in the Netherlands were identified by linking data from the BRP on the officially registered relationships and the sex of people in the Netherlands. Same-sex couples in the Netherlands can officially register their partnership from January 1, 1998 onwards, while same-sex marriage became legal on April 1, 2001. Therefore, all individuals who started an official relationship (i.e., marriage or registered partnership) in the Netherlands since January 1, 1998, were included in the sample. This resulted in a total sample of 3,540,268 individuals, among which 75,362 individuals (2.1%) who had been in a same-sex relationship at least once (including 1,788 individuals who had been in an officially registered relationship with both a male and a female) and 3,464,906 individuals (97.9%) who were only married or had a registered partnership with someone from the opposite sex.

### Measures

Information on criminal behavior was obtained via police registered suspect data from Statistics Netherlands, which were available from 1996 onwards. These police registrations include information about how often individuals in the Netherlands received a “procès-verbal” (i.e., an official report drawn up by police officers about an offense) for various types of crime, in each year between 1996 and 2020. Offenses were only registered when suspects had been charged with an offense of sufficient seriousness that it was eligible for prosecution in the justice system. An important point of consideration, however, is that individuals issued a “procès-verbal” are not, by definition, convicted of a crime. This represents an important methodological consideration. Besjes and Van Gaalen (2008) offered some additional useful insights in regard to what typically follows from a “procès-verbal”, suggesting that over 90 percent of suspects issued one were also found guilty by a judge, or received a transaction (e.g., a fine; see also van de Weijer & Boutwell, 2022). For each person, a binary variable was constructed indicating whether he or she had been suspected of committing a crime at least once within the given time period. In total, 14.52% of all individuals in the sample had been a suspect at least once between 1996 and 2020.

Six additional dichotomous variables were constructed indicating whether individuals had been a suspect of the following types of crime, between 1996 and 2020: violent offenses (prevalence: 5.38%), property offenses (4.82%), vandalism and public order (3.21%), traffic offenses (5.64%), drugs offenses (1.35%), and other offenses (1.97%).^3^ For additional sensitivity analyses, the same variables were constructed based on court data which contain all criminal cases that were registered by the Dutch public prosecutor’s office between 2001 and 2019. In comparison with the police data, the court data exclude the cases in which the police did not find enough evidence against a suspect to send the case to the public prosecutor. It remains, nevertheless, unknown whether prosecuted persons were actually convicted for a given crime. Hence, registered persons should only be considered as suspects of a crime.

The focal independent variable was same-sex or opposite-sex relationship involvement, which was constructed dichotomously to indicate whether an individual had been in an officially registered same-sex relationship at least once or only had been in opposite-sex relationships. Information on the registered relationship and sex of the sample members enabled us to compare men in opposite-sex relationships (*N* = 1,729,743; 48.86%), men in same-sex relationships (*N* = 36,752; 1.04%), women in opposite-sex relationships (*N* = 1,735,163; 49.01%), and women in same-sex relationships (*N* = 38,610; 1.09%).

The 1788 individuals who had been in an officially registered relationship with both men and women were considered as being in a same-sex relationship. We did not construct separate categories for bisexual men and women because these groups would be relatively small for the varieties of analyses used in this study. It was, moreover, not possible to identify bisexual individuals who only had one officially registered relationship.

Four control variables were included in the analyses. First, year of birth (M: 1973.56; median: 1975; mode: 1972) was derived from the BRP and included since people from older birth cohorts may have been less likely to be in a same-sex relationship, while younger people were probably more likely to offend between 1996 and 2020.

Second, the data from the BRP were also used to construct a variable indicating the migration background of sample members. This variable was included since people with non-Western migration backgrounds may have been less likely to be in a same-sex relationship than Dutch natives, due to religious and cultural differences between both groups. Non-Western individuals have also been found to have a considerably higher arrest risk (Boon et al., 2019). Three groups were distinguished based on the country of birth of the sample members and their parents: Dutch natives (i.e., the sample member and both parents were born in the Netherlands; 71.45%), Western immigration background (i.e., the sample member and/or parents were born in Europe [excluding Turkey], North America, Oceania, Japan or Indonesia; 11.47%), and non-Western immigration background (i.e., the sample member and/or parents were born in Africa, Asia [excluding Indonesia and Japan], Latin America, or Turkey; 17.08%).^4^

Third, the data from the BRP were used to construct an exposure variable which indicates in how many years between 1996 and 2020 a sample member was registered as living in the Netherlands (M 23.65; SD 3.13), since the current study could only take into account observed crimes committed in the Netherlands. The years in which a sample member lived abroad or after he or she deceased were excluded. For the sensitivity analyses based on court data, a separate exposure variable was constructed indicating the number of years sample members were registered as living in the Netherlands between 2001 and 2019.

Fourth, sample members’ highest obtained educational level was included as a control variable as studies have shown that men and women in same-sex relationships have higher educational levels than those in opposite-sex relationships (e.g., Andersson et al., 2006) and that educational attainment is negatively related to criminal behavior (e.g., Lochner & Moretti, 2004). Data on sample members’ educational level were obtained from various sources within the System of Social Statistical Files (in Dutch: Stelsel van Sociaal-statistische Bestanden; SSB). Following the standard classification of Statistics Netherlands, three categories were distinguished: low (i.e., primary and lower secondary education; 10.26%), medium (i.e., higher secondary education; 24.89%), and high (i.e., tertiary education; 29.97%) educational level. These data were available for everyone living in the Netherlands from 1999 onwards although coverage is not complete, particularly not for individuals from older birth cohorts. This resulted in 1,234,994 (34.88%) missing values. Multiple imputation was therefore used in the regression models to handle the missing values on educational level in the regression analyses.

### Statistical Analyses

Bivariate analyses were first used to compare the crime rates between men in same-sex and opposite-sex relationships, as well as between women in same-sex and opposite-sex relationships. Odds ratios were used to indicate the relative risk of involvement in criminal behavior. Following these models, we estimated multivariate logistic regression analyses to test whether differences between groups remained significant after controlling for year of birth, migration background, exposure time, and educational level. Finally, the kinship data in the BRP were used to identify siblings within the sample. Discordant sibling models were then used in order to introduce more stringent control over unmeasured familial confounders. In these models, pair-wise comparisons were made between men in same-sex relationships and their brothers in opposite-sex relationships, and between women in same-sex relationships and their sisters in opposite-sex relationships. By comparing within families, the model controls for all environmental confounders shared between siblings (Willoughby et al., 2023).

There is also some correction for confounding from heritable factors, but it is important to be clear that some possibility of genetic confounding persists in these models. To understand why, recall that while full siblings share 50% of their distinguishing genetic material, the remaining portion that differs represents a possible source of spuriousness (see also D’Onofrio et al., 2013). In total, 5355 pairs of discordant brothers (i.e., 10,710 males) and 6513 pairs of discordant sisters (i.e., 13,026 females) could be identified. In these models, only sibling pairs who were also discordant on the crime outcome variables contribute to the regression coefficients.^5^ The effective sample sizes of the estimations are therefore lower than 10,710 and 13,026. All analyses were carried out for different types of crime separately and for the variable indicating any type of criminal behavior.

Sensitivity analyses were conducted for two reasons. First, all analyses were repeated using the court data in order to further scrutinize the robustness of our results. As could be expected, there is much similarity in the variables based on police and court data: 93% of the sample members had the same score, 5.7% was registered as a suspect in the police data but not in the court data, and 1.3% was registered as a suspect in the court data but not in the police data. The results of these sensitivity analyses are presented in Appendix 1.

Second, other sensitivity analyses were carried out since police data were only available from 1996 onwards and sample members may not have been living in the Netherlands until the end of follow-up, in 2020. Although we included a variable indicating exposure time in the main analyses in order to control for such differences, extra analyses were carried out among only those sample members born between 1984 and 1990 and still living in the Netherlands at age 30 (*N* = 425,423). Since for this group the police data are complete between age 12 (i.e., the minimum age of criminal responsibility) and 30, the dependent variable in these sensitivity analyses is a registration in the police data up to age 30. In this smaller group, the number of siblings who were discordant on type of relationship and criminal behavior was very low (i.e., 112 discordant brothers and 92 discordant sisters). Therefore, the discordant siblings model was not estimated for specific types of crimes. The results of these sensitivity analyses can be found in Appendix 2.

All analyses were conducted in Stata version 16.

## Results

**Fig. 1:**
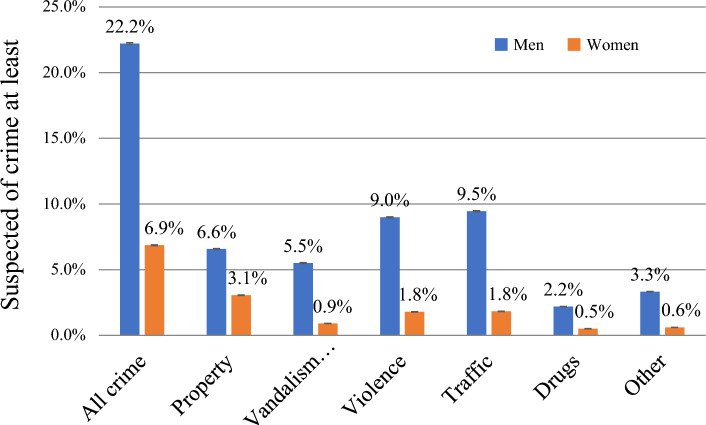
Sex and crime between 1996 and 2020. *Note* N men = 1,766,495; N women = 1,773,773

Figure 1 shows the differences in criminal behavior between men and women for different types of crime. In total, 22.2% of the male sample members were suspected of crime at least once between 1996 and 2020, compared to 6.9% of the women (odds ratio [OR]: 3.85). The same pattern was found among all types of crimes. The relative difference was the largest for vandalism and public order (men: 5.5%, women: 0.9%; OR: 6.41) and the smallest for property offenses (men: 6.6%, women: 3.1%; OR: 2.21).

**Fig. 2:**
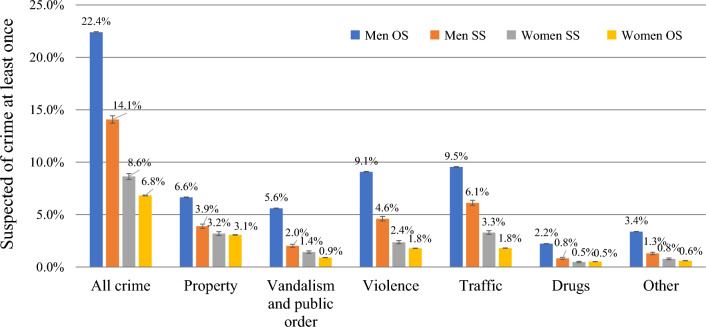
Relationship type and crime between 1996 and 2020. *Note* OS = opposite-sex relationship; SS = same-sex relationship. N men OS = 1,729,743; N men SS = 36,752; N women OS = 1,735,163; N women SS = 38,610

The differences in criminal behavior between men and women in same-sex and opposite-sex relationships are shown in Fig. 2. These comparisons showed that, between 1996 and 2020, men in opposite-sex relationships were most commonly suspected of crime (22.4%), followed by men in same-sex relationships (14.1%), women in same-sex relationships (8.6%), and finally by women in opposite-sex relationships (6.8%). The same pattern was found for all types of crime except for drugs offenses: women in same-sex and opposite-sex relationships were both suspected of a drugs offense in 0.5% of the cases.

**Fig. 3:**
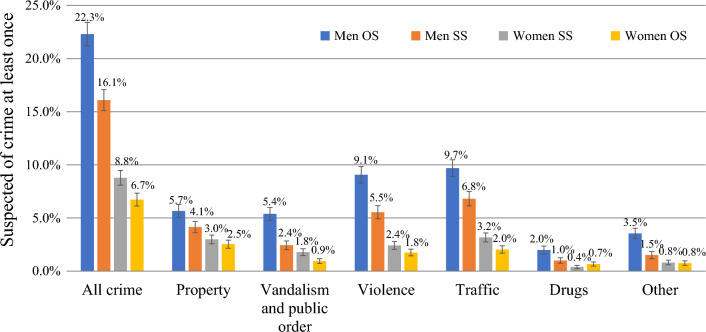
Relationship type and crime between 1996 and 2020, after selection of discordant siblings. *Note* OS = opposite-sex relationship; SS = same-sex relationship. N men OS = 5358; N men SS = 5358; N women OS = 6513; N women SS = 6513

Figure 3 shows the differences in criminal behavior between men and women in same-sex and opposite-sex relationships, after selecting only pairs of siblings who were discordant for type of relationship. The patterns in Fig. 3 align well with those from the total sample displayed in Fig. 2, with the only exception that women in this sub-sample are slightly more likely to commit drug offenses when they are in an opposite-sex relationship (0.7%) than those in a same-sex relationship (0.4%). The fact that the results were very similar after selecting pairs of siblings suggests that accounting for familial factors does not affect differences between individuals in same-sex and opposite-sex relationships much. This will be tested more thoroughly in the discordant sibling analyses in Tables 1, 2, 3, and 4.

**Table 1:** Associations between relationship type of men and crime (full models)

	Bivariate analysis	Multivariate analysis	Discordant sibling model
	OR (95% CI)	OR (95% CI)	OR (95% CI)
Same-sex relationship	0.567 (0.551–0.584)**	0.685 (0.664–0.707)***	0.676 (0.603–0.759)***
*Control variables*			
Year of birth		1.014 (1.014–1.015)***	1.011 (0.990–1.033)
*Migration background:*			
Dutch		Ref. cat	
Western		0.809 (0.798-0.820)***	
Non-western		1.486 (1.471–1.500)***	
*Educational level*			
Low		Ref. cat	Ref. cat
Medium		0.566 (0.559–0.573)***	0.567 (0.446–0.720)***
High		0.217 (0.214–0.220)***	0.290 (0.219–0.384)***
Exposure time		1.056 (1.054–1.057)***	1.042 (1.004–1.081)*
*N*	1,766,495	1,766,495	2918

*p < 0.05, **p < 0.01, ***p < 0.001

**Table 2:** Associations between relationship type of men and different types of crime

	Bivariate analyses	Multivariate analyses	Discordant sibling models
	OR (95% CI)	OR (95% CI)	OR (95% CI)
Property offenses	0.569 (0.539–600)***	0.782 (0.740–0.825)***	0.755 (0.612–0.931)**
Vandalism & public order	0.349 (0.325–0.367)***	0.524 (0.487–0.564)***	0.464 (0.357–0.603)***
Violence	0.483 (0.460–0.507)***	0.601 (0.572–0.632)***	0.605 (0.510–0.719)***
Traffic	0.618 (0.592–0.645)***	0.679 (0.650–0.709)***	0.720 (0.619–0.838)***
Drugs	0.369 (0.329–0.413)***	0.504 (0.450–0.565)***	0.533 (0.359–0.795)**
Other	0.375 (0.343–0.411)***	0.485 (0.443–0.532)***	0.474 (0.344–0.652)***

All rows indicate separate models; *p < 0.05, **p < 0.01, ***p < 0.001

As shown in the bivariate analyses in Tables 1 and 2, all differences between men in opposite-sex and same-sex relationships were significant. The strongest association was found for vandalism and public order (OR: 0.349) while the relationship was the least strong for traffic offenses (OR: 0.618). The bivariate analyses in Tables 3 and 4 show that the differences between women in same-sex and opposite-sex relationships were significant for any crime (OR: 1.290) and specifically for traffic offenses (OR: 1.855), vandalism and public order offenses (OR: 1.594), violence (OR: 1.339), and other offenses (OR: 1.275) No significant differences were found for property and drugs offenses. Additional analyses (not shown in Tables 1, 2, 3, and 4) show that both male groups were significantly more often suspected of committing both any crime, as well as of all separate types of crime, than both female groups.

**Table 3:** Associations between relationship type of women and crime (full model)

	Bivariate analysis	Multivariate analysis	Discordant sibling model
	OR (95% CI)	OR (95% CI)	OR (95% CI)
Same-sex relationship	1.290 (1.245–1.337)**	1.560(1.502–1.620)***	1.550 (1.326–1.813)***
*Control variables*			
Year of birth		1.008 (1.007–1.008)***	1.016 (0.986–1.046)
*Migration background:*			
Dutch		Ref. cat	
Western		0.906 (0.888–0.924)***	
Non-western		1.308 (1.289–1.328)***	
*Educational level*			
Low		Ref. cat	Ref. cat
Medium		0.536 (0.527–0.544)***	0.632 (0.449–0.890)**
High		0.205 (0.201–0.210)***	0.244 (0.158–0.377)***
Exposure time		1.049 (1.047–1.051)***	1.028 (0.970–1.088)
*N*	1,773,773	1,773,773	1644

*p < 0.05, **p < 0.01, ***p < 0.001

**Table 4:** Associations between relationship type of women and different types of crime

	Bivariate analyses	Multivariate analyses	Discordant sibling model
	OR (95% CI)	OR (95% CI)	OR (95% CI)
Property offenses	1.044 (0.986–1.106)	1.390 (1.310–1.475)***	1.461 (1.121–1.905)**
Vandalism & public order	1.594 (1.463–1.737)***	2.144 (1.965–2.340)***	2.323 (1.597–3.379)***
Violence	1.339 (1.253–1.432)***	1.819 (1.697–1.945)***	1.548 (1.161–2.064)**
Traffic	1.855 (1.752–1.965)***	1.707 (1.611–1.809)***	1.695 (0.1335–2.152)***
Drugs	0.930 (0.803–1.077)	1.130 (0.974–1.311)	0.484 (0.228–1.029)
Other	1.275 (1.135–1.431)***	1.440 (1.280–1.619)***	1.108 (0.728–1.686)

All rows indicate separate models; *p < 0.05, **p < 0.01, ***p < 0.001

Multivariate logistic regression analyses were used to test whether these associations would remain significant after controlling for year of birth, migration background, exposure time, and educational level. The multivariate regression estimates in Table 1 are in line with the results in Fig. 2, showing that men in same-sex relationships have significantly lower odds of being a crime suspect than men in opposite-sex relationships (OR = 0.685), including lower odds for being a suspect of property, vandalism and public order, violence, traffic, drugs, and other offenses (Table 2). All associations, however, were weaker than in the bivariate comparisons.

Next, discordant sibling models were used to control for sources of familial confounding shared by siblings. The third model in Table 1 depicts the results of the within-pair estimates for discordant brothers and shows that the odds ratio is very similar to that of the multivariate analyses. Men in same-sex relationships, thus, have a lower risk of being suspected of crime than their brothers in opposite-sex relationships (OR = 0.676). The results of the discordant sibling models in Table 2 show that the odds ratios for the different types of crime are also very similar to those from the multivariate analyses. This suggests that unmeasured familial factors may not fully confound the relationship between same-sex relationships and criminal behavior.

The second model in Table 3 shows the results of the logistic regression analysis in which the relationship between arrests and the type of relationship of women was examined, while controlling for birth year, migration background, exposure time, and educational level. The results are in line with the results in Fig. 2 and indicate a significant association between the type of relationship of women and offending. Women in a same-sex relationship have significantly higher odds of being involved in crime compared to those in an opposite-sex relationship (OR = 1.560). Contrary to the results for men, the odds ratio in the multivariate model is larger compared to the bivariate analyses.

In Table 4, a distinction is made between different types of crime and it is shown that, among women, being in a same-sex relationship is significantly associated with a higher risk for involvement in property crime, vandalism and public order, violence, traffic, and other offenses, but not for drugs offenses. The associations with property offenses, violence, vandalism and public order, and other offenses were stronger in the multivariate models compared to the bivariate models, while the relationship with traffic offenses became weaker.

In additional analyses, not shown in Tables 1, 2, 3, and 4, we also compared the crime rates of all four groups of men and women in same-sex and opposite-sex relationships in multivariate analyses. In all of these analyses, both male groups have significantly higher crime rates than both female groups. Moreover, multivariate regression models were estimated among the total sample which included the control variables, gender, relationship type, and an interaction variable between gender and relationship type. In all these models, a significant interaction was found indicating that the associations between having a same-sex relationship and different types of criminal behavior were in opposite directions for men and women.

The third model in Table 3 reports the results of the discordant sibling models for women. The results show that women in a same-sex relationship have a significantly higher risk of being arrested than their sisters in an opposite-sex relationship (OR: 1.550). The odds ratio from the discordant sibling model was very similar to the odds ratio from the multivariate regression analyses, which suggests that the relationship is robust against some amount of familial confounding. The results in Table 4 show similar results for property crime, vandalism and public order, violence, and traffic offenses. No significant association between type of relationship and other offenses was found anymore in the discordant sibling models. However, the effective sample sizes for the discordant sibling models on drugs offenses (*n* = 96) and other offenses (*n* = 184) were small, and additional caution is prudent for these particular findings.

In order to gauge the extent to which our results were robust, all analyses were repeated with dependent variables based on the court data. The results of these analyses can be found in Appendix 1 and yielded conclusions that were substantively similar to those based on police register suspect data. The most important difference between the results reported in Appendix 1 and those in Tables 1, 2, 3, and 4 is that no significant associations were found between type of relationship and property offenses in the discordant sibling models, for both men and women, when court data was used (see Tables 6 and 8). In addition, the results from the multivariate analyses in Table 8 show that women in a same-sex relationship were significantly more likely to be brought to court for drugs offenses than those in an opposite-sex relationship, while this relationship was not significant in the main analyses.

The results of the sensitivity analyses, in which only sample members born between 1984 and 1990 were included, are presented in Appendix 2. These analyses also yielded in substantively similar conclusions as the main analyses. The only difference among male sample members was that no significant association was found between relationship type and property crime in the multivariate analyses presented in Table 10. Among women, the discordant sibling model in Table 11 did not show a significant relationship between relationship type and criminal offending. However, since the odds ratio of this discordant sibling model was higher than that of the multivariate analyses, this insignificant association seems to be the result of the decreased statistical power in this sensitivity analyses rather than of controlling for unmeasured familial confounders.

## Discussion

This study examined the relationship between same-sex relationships and criminal behavior using longitudinal register data from the entire population of the Netherlands. The results of the study suggested that men in opposite-sex relationships were more often suspected of crime than were men in same-sex relationships, while women in opposite-sex relationships were less often suspected of crime than women in same-sex relationships. The disproportionate involvements of same-sex relationship males and females in criminal outcomes are unsurprising and accord with decades of scholarship (e.g., Beaver et al., 2016; Jonnson et al., 2019; Pinhey & Brown, 2005; Sergeant et al., 2006). One interesting divergence, however, was that individuals identifying as lesbians reported more antisocial behavior than gay males in the Beaver et al. (2016) study, a finding not replicated here. Moreover, the meta-analysis of Jonnson et al. (2019) did not show a lower risk for sexual minority boys in terms of juvenile justice system involvement, while this was consistently found in the current study.

Our results seem in line with studies finding sexual minorities to be “shifted” in a cross-sex direction (i.e., gay men being somewhat behaviorally “feminized” and lesbians being somewhat “masculinized” compared to their heterosexual counterparts), for example in their aggressiveness (Ellis et al., 1990) and delinquent behavior (Beaver et al., 2016). This pattern is in line with biologically informed theories, such as Ellis and Ames’ (1987) prenatal androgen theory, which explain the association between sexual minority status and behavior as resulting from prenatal testosterone exposure differences emergent between gay males and lesbians compared to heterosexual individuals. Measures of prenatal testosterone exposure were unavailable here, but as we mentioned earlier, the generally tenuous associations between prenatal testosterone and antisocial behaviors poses a theoretical problem for these particular perspectives.

The minority stress model also seems to be limited in key respects for explaining all the patterns observed here. Although stigma, prejudice, and discrimination can create a hostile and stressful social environment for sexual minorities, which in turn could result in adverse outcomes (Lick et al., 2013; Meyer, 2003), only the offending patterns of women seems to accord with the minority stress model. For males the findings appear incongruous. It seems reasonable to assert that men in same-sex relationships are exposed in similar ways to prejudicial treatment as women in same-sex relationships. Assuming this exposure has *some* influence on crime, it seems to be that of driving down the criminal behavior of these men, not up.

An important novel finding of the current study is that the associations between same-sex relationships and offending were not only found for crime in general, but for specific types of crimes as well. Men in same-sex relationships were less likely to be suspected of any type of crime (i.e., property offenses, vandalism and public order, violence, traffic offenses, drug offenses, and other offenses) than men in opposite-sex relationships. Among the women, those in same-sex relationships were more likely to be suspected of any type of crime than those in opposite-sex relationships, except for drug offenses. These results indicate that our findings are generalizable to a wide array of criminal behaviors.

Another important novel finding of this study is that the association between same-sex relationships and criminal offending could not be fully explained by unobserved factors shared by siblings. Since previous studies found that both sexual orientation (Bailey & Pillard, 1995; Cook, 2021; Kendler et al., 2000; Kirk et al., 2000) and antisocial behaviors (Ferguson, 2010; Mason & Frick, 1994; Rhee & Waldman, 2002) are partly heritable, it is important to use methods that can correct for these factors. The discordant sibling models used here compared members in same-sex relationships to their full siblings in opposite-sex relationships. This phase of the analysis could account for all shared environmental sources of spuriousness and partly for those attributable to heritable influences. The outcome was largely the same as the results calculated from the total population. At least in these data, it seems that correction for shared environmental factors (and partial correction for genetic factors) could not fully account for the association between same-sex relationships and offending.

By the nature of what the models are doing, the persistence of the association suggests that the differences in experience for same-sex versus opposite-sex union individuals directly impacts their risk of antisocial behaviors. Identifying the precise nature of the pathways will hopefully be a goal in future work. In terms of plausible possibilities, educational attainment is an example of a variable with far-reaching implications for numerous outcomes, crime included (e.g., Lochner & Moretti, 2004), while studies have also shown that people in same-sex marriages have higher average educational levels than those in opposite-sex marriages (e.g., Andersson et al., 2006). In the case of our study, the overall conclusions remained unaltered after controlling for educational attainment, suggesting that at the moment it does not (fully) explain the association between same-sex relationship and criminal offending. It will be interesting to see what possibilities emerge in future research.

Despite some desirable qualities, our study possessed some key limitations that warrant discussion. First, we used official justice system data to measure criminal behaviors as opposed to self-reported behaviors. Some amount of error owing to this strategy is likely, as it is reasonably well known that official registrations underestimate the true prevalence of crime (Biderman & Reiss, 2017). To the extent that those in same-sex relationships are more or less likely to get arrested by law enforcement, this would suggest that the results of this study are biased in some direction. The sensitivity analyses based on court data and prior research of Beaver et al., (2016) using self-reported data, however, produced similar substantive conclusions, but additional work is still needed. Another limitation of the data used here is that they were only available from 1996 onwards and, thus, may be incomplete for the older sample members. However, this did not seem to have a large impact on our findings since similar results were found in sensitivity analyses among only those sample members for whom information about criminal offending was complete.

Returning to a point we raised early in the paper, we lacked an actual measure of self-reported sexual orientation, and instead relied on official statistics documenting same-sex relationships. This permitted us to make only broad distinctions between men and women in same-sex or opposite-sex relationships. Bisexual men and women, for example, could not clearly be identified and examined as a separate category. Bisexual individuals have been previously found to display particularly elevated levels of aggression (Ellis et al., 1990) and delinquent behavior (Beaver et al., 2016). Whether this produced an underestimation or overestimation of the associations found here is impossible to determine, however, since we do not know whether bisexual individuals were more often in our same-sex relationship or opposite-sex relationship groups.

Besides bisexuality, sexual orientation and gender identity are considerably more nuanced than what is reflected in our categorical variable, and individual sexuality might be relatively fluid as opposed to a stable construct. Our ability to adequately model either fluid or static qualities pertaining to sexuality was strongly limited. Registration data of this type simply do not provide an ability to account for these important issues. Future studies should therefore aspire to use large samples with self-reported data capable of grasping the considerable complexities of both gender identity and sexual orientation. Another limitation of measuring sexual orientation status by proxy with officially registered relationships is that only individuals who were ever married or had a registered partnership end up being included in the analytical sample. Since criminological research has suggested that married individuals report less criminal and antisocial behavior, in general, than their non-married counterparts (Siennick & Osgood, 2008), the prevalence of criminal behavior could be lower overall in our study. A selection bias of this kind might function to attenuate some of the effects we observed here, as more criminogenic individuals would be missing. However, if gay males are less involved in crime than heterosexual males, regardless of marital status, the effects of this particular selection bias become a bit more complex.

Considered in the context of the limitations we just described, the current study represents one of the first (if not *the* first) attempts to examine the association between sexual preference and offending outside of North America using a population that differs notably in national attitudes about sexual preference. Data drawn from the entire Dutch population afforded us the capability of distinguishing between different types of criminal behavior and also to utilize siblings in order to more rigorously account for certain types of confounders that are virtually impossible to fully account for in standard social science models (Barnes et al., 2014; Willoughby et al., 2023). This study extends existing knowledge related to sexual preference and antisocial outcomes, showing that men in a same-sex relationship are less likely, and women in a same-sex relationship are more likely, to be suspected of essentially all forms of crime compared to their counter parts in opposite-sex relationships. Illuminating the chains of causation which explain our results remains one of the key challenges for future theoretical and empirical work.

Finally, it is worth reiterating that genetically informed research designs offer interesting opportunities for improved causal inference around this topic when only non-experimental data are available, as was recently illustrated by Oginni et al. (2023a, b). Using Mendelian randomization-direction of causation models to examine the effects of same-sex attraction on psychological distress and risky sexual behavior, the results suggested that associations between sexual orientation and distress may indeed reflect causal effects of one on the other (with influences perhaps running in both directions; Oginni et al., 2023b). Beyond a particular finding, though, the results further illustrate the usefulness of genetically sensitive designs to explore environmental influences on human outcomes (Barnes et al., 2014; Willoughby et al., 2023).

## Data Availability

Data and materials are not publicly available since these are stored on the secure servers of Statistics Netherlands.

## Appendix 1: Analyses Based on Court Data

See Tables 5, 6, 7, and 8

**Table 5:** Associations between relationship type of men and crime (measured with court data)

	Bivariate analysis	Multivariate analysis	Discordant sibling model
	OR (95% CI)	OR (95% CI)	OR (95% CI)
Same-sex relationship	0.537 (0.517–0.556)***	0.670 (0.646–0.696)***	0.633 (0.552–0.728)***
*Control variables*			
Year of birth		1.019 (1.018–1.019)***	1.002 (0.977–1.027)
*Migration background:*			
Dutch		Ref. cat	
Western		0.829 (0.816–0.842)***	
Non-western		1.585 (1.568–1.602)***	
*Educational level*			
Low		Ref. cat	Ref. cat
Medium		0.548 (0.540–0.553)***	0.569 (0.402–0.807)**
High		0.200 (0.196–0.203)***	0.230 (0.153–0.345)***
Exposure time		1.083 (1.081–1.085)***	1.068 (1.013–1.127)*
*N*	1,766,495	1,766,495	2216

**p* < 0.05, ***p* < 0.01, ****p* < 0.001

**Table 6:** Associations between relationship type of men and different types of crime (measured with court data)

	Bivariate analyses	Multivariate analyses	Discordant sibling models
	OR (95% CI)	OR (95% CI)	OR (95% CI)
Property offenses	0.572 (0.534–0.613)***	0.810 (0.756–0.869)***	0.879 (0.676–1.142)
Vandalism & public order	0.337 (0.305–0.371)***	0.543 (0.492–0.599)***	0.524 (0.378–0.725)***
Violence	0.486 (0.456–0.517)***	0.604 (0.567–0.644)***	0.548 (0.443–0.678)***
Traffic	0.569 (0.536–0.604)***	0.665 (0.626–0.707)***	0.679 (0.544–0.847)***
Drugs	0.352 (0.307–0.403)***	0.482 (0.421–0.552)***	0.451 (0.277–0.732)**
Other	0.465 (0.429–0.504)***	0.581 (0.536–0.629)***	0.699 (0.528–0.925)*

All rows indicate separate models; **p* < 0.05, ***p* < 0.01, ****p* < 0.001

**Table 7:** Associations between relationship type of women and crime (measured with court data)

	Bivariate analysis	Multivariate analysis	Discordant sibling model
	OR (95% CI)	OR (95% CI)	OR (95% CI)
Same-sex relationship	1.280 (1.225–1.338)***	1.619 (1.547–1.695)***	1.575 (1.302–1.904)***
*Control variables*			
Year of birth		1.007 (1.007–1.008)***	1.001 (0.967–1.065)
*Migration background:*			
Dutch		Ref. cat	
Western		0.967 (0.944–0.990)**	
Non-western		1.478 (1.452–1.505)***	
*Educational level*			
Low		Ref. cat	Ref. cat
Medium		0.500 (0.490–0.510)***	0.736 (0.489–1.109)
High		0.187 (0.182–0.192)***	0.280 (0.165–0.473)***
Exposure time		1.093 (1.089–1.097)***	0.982 (0.905–1.065)
*N*	1,773,773	1,773,773	1048

**p* < 0.05, ***p* < 0.01, ****p* < 0.001

**Table 8:** Associations between relationship type of women and different types of crime (measured with court data)

	Bivariate analyses	Multivariate analyses	Discordant sibling model
	OR (95% CI)	OR (95% CI)	OR (95% CI)
Property offenses	1.005 (0.931–1.083)	1.417 (1.312–1.531)***	1.379 (0.987–1.926)
Vandalism & public order	1.455 (1.295–1.634)***	2.157 (1.917–2.428)***	1.639 (1.008–2.664)*
Violence	1.385 (1.267–1.514)***	1.856 (1.694–2.033)***	1.782 (1.191–2.665)**
Traffic	1.911 (1.762–2.072)***	1.821 (1.677–1.977)***	2.202 (1.505–3.222)***
Drugs	1.023 (0.866–1.209)	1.236 (1.044–1.464)*	0.739 (0.375–1.458)
Other	1.371 (1.238–1.517)***	1.586 (1.431–1.759)***	1.362 (0.873–2.125)

All rows indicate separate models; **p* < 0.05, ***p* < 0.01, ****p* < 0.001

## Appendix 2: Analyses among Sample Members Born Between 1984 and 1990

See Tables 9, 10, 11, and 12

**Table 9:** Associations between relationship type of men and crime (only sample members born 1984–1990)

	Bivariate analysis	Multivariate analysis	Discordant sibling model
	OR (95% CI)	OR (95% CI)	OR (95% CI)
Same-sex relationship	0.529 (0.466–0.560)***	0.578 (0.507–0.659)***	0.518 (0.275–0.973)*
*Control variables*			
Year of birth		1.000 (0.994–1.006)	1.085 (0.866–1.359)
*Migration background:*			
Dutch		Ref. cat	
Western		1.359 (1.285–1.437)***	
Non-western		3.128 (3.031–3.227)***	
*Educational level*			
Low		Ref. cat	Ref. cat
Medium		0.423 (0.408–0.439)***	0.554 (0.153–2.003)
High		0.146 (0.141–0.152)***	0.194 (0.043–0.881)*
*N*	182,392	182,392	112

**p* < 0.05, ***p* < 0.01, ****p* < 0.001

**Table 10:** Associations between relationship type of men and different types of crime (only sample members born 1984–1990)

	Bivariate analyses	Multivariate analyses
	OR (95% CI)	OR (95% CI)
Property offenses	0.669 (0.550–0.813)***	0.833 (0.681–1.018)
Vandalism & public order	0.326 (0.256–0.416)***	0.350 (0.274–0.447)***
Violence	0.527 (0.431–0.644)***	0.613 (0.499–0.752)***
Traffic	0.511 (0.413–0.632)***	0.566 (0.457–0.701)***
Drugs	0.486 (0.319–0.741)***	0.620 (0.405–0.948)*
Other	0.363 (0.255–0.519)***	0.417 (0.291–0.596)***

All rows indicate separate models; **p* < 0.05, ***p* < 0.01, ****p* < 0.001

**Table 11:** Associations between relationship type of women and crime (only sample members born 1984–1990)

	Bivariate analysis	Multivariate analysis	Discordant sibling model
	OR (95% CI)	OR (95% CI)	OR (95% CI)
Same-sex relationship	1.585 (1.427–1.760)***	1.836 (1.647–2.046)***	2.085 (0.972–4.470)
*Control variables*			
Year of birth		1.023 (1.015–2.032)***	0.916 (0.690–1.216)
*Migration background:*			
Dutch		Ref. cat	
Western		1.574 (1.461–1.696)***	
Non-western		2.554 (2.455–2.657)***	
*Educational level*			
Low		Ref. cat	Ref. cat
Medium		0.338 (0.324–0.353)***	0.425 (0.087–2.071)
High		0.115 (0.109–0.121)***	0.023 (0.001–0.0375)**
*N*	242,491	242,491	92

**p* < 0.05, ***p* < 0.01, ****p* < 0.001

**Table 12:** Associations between relationship type of women and different types of crime (only sample members born 1984–1990)

	Bivariate analyses	Multivariate analyses
	OR (95% CI)	OR (95% CI)
Property offenses	1.366 (1.163–1.603)***	1.626 (1.379–1.916)***
Vandalism & public order	2.146 (1.762–2.613)***	2.403 (1.966–2.936)***
Violence	1.802 (1.518–2.141)***	2.085 (1.749–2.485)***
Traffic	1.737 (1.415–2.133)***	1.857 (1.511–2.281)***
Drugs	1.394 (0.929–2.091)	1.501 (0.998–2.257)
Other	1.876 (1.383–2.544)***	2.096 (1.541–2.851)***

All rows indicate separate models; **p* < 0.05, ***p* < 0.01, ****p* < 0.001

## Footnotes

1 It is important to note that if familial factors confound the relationship between same-sex relationships and offending, the discordant siblings model will not provide insights in whether it was confounded by genetic or shared environmental factors. In order to draw conclusions about the source of confounding, a sample with pairs of dizygotic and monozygotic twins is necessary (see also D’Onofrio et al., 2013; Gonggrijp et al., 2023).

2 Under certain conditions, these microdata sets are accessible for statistical and scientific research (for further information: microdata@cbs.nl).

3 Other offenses include all offenses which did not fit in the other categories. These include, for example, perjury, poaching, violations of local ordinances, and environmental crimes.

4 Individuals with an immigration background from Japan or Indonesia were considered as having a Western immigration background based on their social-economic and social-cultural position. These are mainly people who (or whose parents) were born in former Dutch East Indies or employees of Japanese companies.

5 The other pairs of siblings either both offended or both abstained from offending. Although these are perfectly valid observations, they do not contribute to the estimation due to a lack of within-pair variation (Gonggrijp et al., 2023) and were therefore not included in the effective sample sizes of the estimation.
